# A comparative study on the volume and localization of the internal gross target volume defined using the seroma and surgical clips based on 4DCT scan for external-beam partial breast irradiation after breast conserving surgery

**DOI:** 10.1186/1748-717X-9-76

**Published:** 2014-03-19

**Authors:** Yun Ding, Jianbin Li, Wei Wang, Suzhen Wang, Jinzhi Wang, Zhifang Ma, Qian Shao, Min Xu

**Affiliations:** 1 Department of Radiation Oncology (Chest section), Shandong Tumor Hospital, Jinan, Shandong Province, 250117, People’s Republic of China; 2 Medicine and life sciences college of Shandong Academy of Medical Sciences, Jinan University, Jinan, Shandong Province 250200, People’s Republic of China

**Keywords:** Four-dimensional computed tomography, Surgical clips, Seroma, Internal gross target volume

## Abstract

**Background:**

To explore the volume and localization of the internal gross target volume defined using the seroma and/or surgical clips based on the four-dimensional computed tomography (4DCT) during free-breathing.

**Methods:**

Fifteen breast cancer patients after breast-conserving surgery (BCS) were recruited for EB-PBI. On the ten sets CT images, the gross target volume formed by the clips, the seroma, both the clips and seroma delineated by one radiation oncologist and defined as GTVc, GTVs and GTVc + s, respectively. The ten GTVc, GTVs and GTVc + s on the ten sets CT images produced the IGTVc, IGTVs, IGTVc + s, respectively. The IGTV volume and the distance between the center of IGTVc, IGTVs, IGTVc + s were all recorded. Conformity index (CI), degree of inclusion (DI) were calculated for IGTV/IGTV, respectively.

**Results:**

The volume of IGTVc + s were significantly larger than the IGTVc and IGTVs (p < 0.05). There was significant difference between the DIs of IGTVc vs IGTVc + s, the DIs of IGTVs vs IGTVc + s. There was significant difference among the CIs of IGTV/IGTV. The DIs and CIs of IGTV/IGTV were negatively correlated with their centroid distance (r < 0, p < 0.05).

**Conclusions:**

There were volume difference and spatial mismatch between the IGTVs delineated based on the surgical clips and seroma. The IGTV defined as the seroma and surgical clips provided the best overall representation of the ‘true’ moving GTV.

## Background

Breast-conserving surgery (BCS) followed by whole breast radiotherapy (RT) is the standard treatment for early stage breast cancer [1,2]. Over the past several years, several studies have reported that most of the local recurrences occurred in the vicinity of the tumor bed (TB) for the patients accepted BCS [2,3]. Several researches illustrated that partial breast irradiation (PBI) delivers a radiation therapy to the postoperative TB with a margin of adjacent breast tissue can achieve excellent results after strict patient selection and accurate target volume delineation, and external-beam partial breast irradiation (EB-PBI) is an important approach in PBI [4,5].

The important components for EB-PBI were the TB delineation and displacement measurement [6,7]. Previous studies have reported that seroma and surgical clips were important surrogates in the TB delineation [7,8]. The residual error was no more than 5 mm after on-line error correction based on cone-beam CT (CBCT) [9]. The intrafractional motion was the major contribution for the geometric expansion of the gross tumor volume (GTV) for conventional three-dimensional computed tomography (3DCT) and intensity modulated radiotherapy (IMRT) treatment planning. The internal gross target volume (IGTV) incorporating the intrafraction motion of the GTV has been adopted in many literatures [10-12], in view of the definition of internal target volume (ITV) in the International Commission on Radiation Units and Measurements report 62. Four-dimensional CT (4DCT) scans can capture intrafractional TB mobility for radiotherapy planning and generate accurate IGTV [13].

The distance between the surgical clips and edge of CT-defined seroma in a coronal plan has been investigated in previous study [13], and the displacements of the target volume delineated based on clips and seroma have been obtained using 4DCT in prior studies [14-16]. The volume difference and relative position among the IGTVs defined using the seroma and/or surgical clips have not been fully studied. In the current study, we initially defined three IGTVs using the seroma and/or surgical clips for EB-PBI on 4DCT. In addition, the variations in target size, position, degree of inclusion (DI) [17] and conformity index (CI) [18] were compared. The aim of the study is to investigate the use of 4DCT in the individual IGTV definition of EB-PBI.

## Methods

### Patients

Institutional Review Board, Shandong Tumor Hospital Ethics Committee approval and informed consent were obtained for the present study. The study population consisted of 15 consecutive breast cancer patients (7 left-sided and 8 right-sided lesions) with early-stage breast cancer referred to postoperative RT after BCS. The average duration between lumpectomy and start of radiotherapy planning was 75 days (range, 26–126). For every patient, more than four surgical clips were used to mark the boundaries of the lumpectomy cavity. In order to improve the delineation accuracy, in this study all the patients recruited were with seroma clarity score (SCS) 3 ~ 5 [19] in the lumpectomy cavity. All the patients were free from chronic lung diseases and their ventilation functions were normal, and accepted free-breathing training. The patients enrolled were counseled and signed consent protocol.

### 4D-CT simulation and image acquisition

All the 15 patients underwent a standard free-breathing (FB) virtual CT breast simulation with both arms outreached and raised in a supine position on a breast board. The 4D-CT images recording the respiratory signal were acquired with a thickness of 3 mm at the conclusion of the standard CT simulation using a 16-slice Brilliance big bore CT scanner (Philips Medical Systems, Inc., Cleveland, OH, USA). The signals were sent to the scanner to label a time tag on each CT image. GE Advantage 4D software (GE Healthcare, Waukesha, WI, USA) sorted the reconstructed 4D-CT images into ten respiratory phases on the basis of these tags, with 0% corresponding to end-inhalation (CT_0_) and 50% corresponding to end-exhalation (CT_50_). Then the constructed 4D-CT images sets were transferred to the Eclipse treatment planning system (TPS) (Eclipse 8.6, Varian Medical Systems, Palo Alto, CA, USA) for structure delineation.

### Gross tumor volumes delineation

The 10% ~ 90% phases of 4D-CT images were registered on the 0% phase images, which served as the basic phase image. For each patient, the TB formed by all the clips, seroma, both clips and seroma named as GTVc, GTVs and GTVc + s, respectively. All GTVs were delineated by the same radiation oncologist of the 4D-CT images. The combined volume of the GTVc, GTVs and GTVc + s on the 10 CT phases was defined as internal gross target volume IGTVc, IGTVs and IGTVc + s, respectively. For each patient, the volumes of the IGTVc, IGTVs and IGTVc + s were recorded.

### Centroid distance

For each patient, the three dimensional coordinates of the IGTVc, IGTVs and IGTVc + s were recorded. Then, the displacements between IGTVx and IGTVy in the left-right (LR), anterior-posterior (AP) and superior-inferior (SI) directions were obtained and marked as Δx, Δy and Δz. The distance of the center of mass (COM) between IGTVx and IGTVy were calculated as followed:

$$V={\left({\mathrm{\Delta x}}^{2}+{\mathrm{\Delta y}}^{2}+{\mathrm{\Delta z}}^{2}\right)}^{½}$$

### IGTVs comparison

The volume and degree of inclusion (DI) [17] and degree of conformity index (CI) [18] between IGTVc and IGTVs, IGTVc and IGTVc + s, IGTVs and IGTVc + s were calculated and compared, respectively. The definition of DI of volume A included in volume B (DI (A in B)) was the percentage of the overlap between volume A and B in volume A [17]. The formula was as followed:

$$\mathrm{DI}\left(A\;\mathrm{in}\;B\right)=\frac{A\cap B}{A}$$

Assumed volume B was reference for the standard volume, if the treatment planning was based on volume A, there would be 1-DI (A in B) of volume A being unnecessary irradiated and 1-DI (B in A) of volume B missing irradiation.

The conformity index of volume A and B (CI (A, B)) was computed according to Struikmans *et al*. [18]. The formula was as followed:

$$\mathrm{CI}\left(A,B\right)=\frac{A\cap B}{A\cup B},$$

which is defined as the ratio of the intersection of A with B to the union of A and B. For each patient, the CI and DI were calculated on every phase separately, and then averaged over the 10 phases.

### Statistical analysis

The SPSS 17.0 software was used for statistical analysis. One-Way ANOVA test was used to compare the volume difference among the IGTVs, and the CIs. A paired t test was used for the comparison of DIs. Pearson correlation test was used to study the relationship between the DI, CI and their centroid distance. Statistical significance was defined as a p value of <0.05.

## Results

### The volumes of IGTVs

The volumes of IGTVc, IGTVs and IGTVc + s were listed in Table 1. The volume of IGTVc + s was significantly larger than that of IGTVc and IGTVs (*t* = -2.734, -7.132, *p* = 0.016, 0.000), and there was no significant volume difference between IGTVc and IGTVs (*t* = 1.313, *p* = 0.210).

**Table 1:** **The volumes of IGTVc, IGTVs and IGTVc + s (cm**^**3**^**)**

	**IGTVc**	**IGTVs**	**IGTVc + s**
$\bar{x}\pm s$	28.35 ± 17.54	24.19 ± 21.53	35.73 ± 19.77
95% CI	18.64 ~ 38.06	12.26 ~ 36.11	24.78 ~ 46.67

*Abbreviations*: *IGTV* internal gross tumor volume, *IGTVc* the internal gross tumor volume combined by GTVc, *IGTVs* the internal gross tumor volume combined by GTVs, *IGTVc + s* the internal gross tumor volume combined by GTVc + s.

### The centroid distance between gross tumor volumes

The distance of the COM between IGTVc and IGTVs, IGTVc and IGTVc + s, IGTVs and IGTVc + s were listed in Table 2. The distance of the COM between IGTVc and IGTVs (D-IGTVc/IGTVs) was significant larger than that between IGTVs and IGTVc + s (D-IGTVs/IGTVc + s) (*t* = 3.671, *p* = 0.001).

**Table 2:** The distance of the COM between IGTVs (cm)

	**D-IGTVc/IGTVs**	**D-IGTVc/IGTVc + s**	**D-IGTVs/IGTVc + s**
$\bar{x}\pm s$	0.66 ± 0.31	0.42 ± 0.36	0.32 ± 0.17
95% CI	0.49 ~ 0.83	0.22 ~ 0.61	0.23 ~ 0.42

*Abbreviations*: *D-IGTVc/IGTVs* the distance of the COM between IGTVc and IGTVs, *D-IGTVc/IGTVc + s* the distance of the COM between IGTVc and IGTVc + s, *D-IGTVs/IGTVc + s* the distance of the COM between IGTVs and IGTVc + s.

### DI

**Figure 1:**
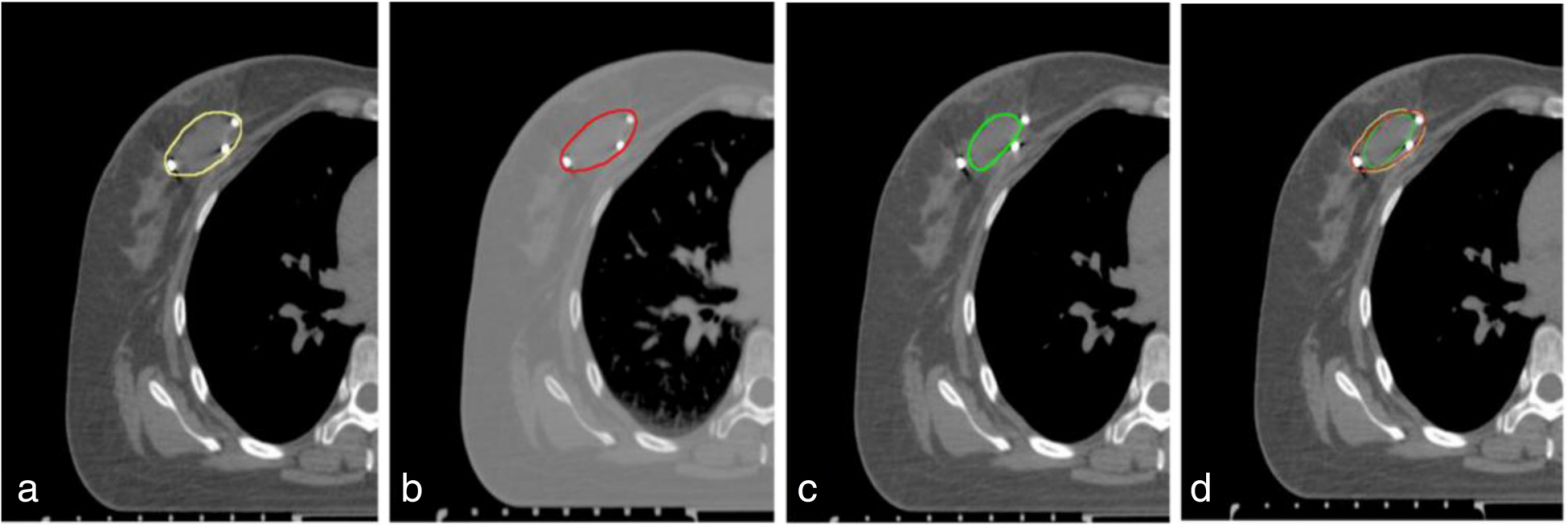
**The outline of GTVc, GTVs and GTVc + s. a**. GTVc + s: the gross target volume delineated based on both seroma and surgical clips; **b**. GTVc: clips the gross target volume delineated based on surgical clips; **c**. GTVs: the gross target volume delineated based on the seroma; **d**. GTVc + s (yellow), GTVc (red) and GTVs (green) on the same lay of a 4D-CT data set.

**Figure 2:**
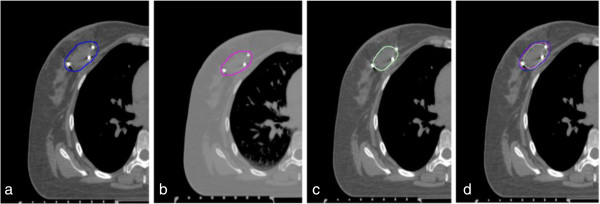
**The outline of IGTVc, IGTVs and IGTVc + s. a**. IGTVc + s: the IGTV combined from GTVc + s; **b**. IGTVc: the IGTV combined from GTVc; **c**. IGTVs: the IGTV combined from GTVs. **d**. IGTVc + s (blue), IGTVc (magenta) and IGTVs (light green) on the same lay of 0% phase images.

Table 3 displayed the DI difference between the three IGTVs delineated based on clips, seroma, both clips and seroma. The DI of IGTVc included in IGTVc + s (DI (IGTVc in IGTVc + s)) was larger than the DI of IGTVc + s included in IGTVc (DI (IGTVc + s in IGTVc)). The DI (IGTVs in IGTVc + s) was larger than the DI (IGTVc + s in IGTVs). The DI (IGTVs in IGTVc), DI (IGTVc + s in IGTVc) and DI (IGTVc + s in IGTVs) were negatively with their center distance (*r* = -0.640, -0.795, -0.576; *p* = 0.010, 0.000, 0.025), respectively. The outline difference among GTVc, GTVs and GTVc + s was shown in Figure 1. The outline difference among IGTVc, IGTVs and IGTVc + s was shown in Figure 2.

**Table 3:** DI comparison of IGTVs delineated based on clips, seroma, both clips and seroma

	**DI (IGTVc in IGTVs)**	**DI (IGTVs in IGTVc)**	**DI (IGTVc in IGTVc + s)**	**DI (IGTVc + s in IGTVc)**	**DI (IGTVs in IGTVc + s)**	**DI (IGTVc + s in IGTVs)**
$\bar{x}\pm s$	0.53 ± 0.20	0.69 ± 0.19	0.88 ± 0.09	0.71 ± 0.18	0.93 ± 0.04	0.57 ± 0.19
t	-2.097		2.976		6.611	
p	0.055		0.010		0.000	

*Abbreviations*: *DI (IGTVc in IGTVs)* the degree of IGTVc included in IGTVs, *DI (IGTVs in IGTVc)* the degree of IGTVs included in IGTVc, *DI (IGTVc in IGTVc + s)* the degree of IGTVc included in IGTVc + s, *DI (IGTVc + s in IGTVc)* the degree of IGTVc + s included in IGTVc, *DI (IGTVs in IGTVc + s)* the degree of IGTVs included in IGTVc + s, *DI (IGTVc + s in IGTVs)* the degree of IGTVc + s included in IGTVs.

### CI

The CI (IGTVc, IGTVc + s) and the CI (IGTVs, IGTVc + s) were larger than the CI (IGTVc, IGTVs) (*t* = -4.367, -2.439; *p* = 0.000, 0.021) (Table 4). The CI (IGTVc, IGTVc + s) and the CI (IGTVs, IGTVc + s) were negatively with their center distance (*r* = -0.822, -0.591; *p* = 0.000, 0.020), respectively.

**Table 4:** The CI between IGTVc, IGTVs and IGTVc + s

	**CI (IGTVc, IGTVs)**	**CI (IGTVc, IGTVc + s)**	**CI (IGTVs, IGTVc + s)**
$\bar{x}\pm s$	0.40 ± 0.14	0.63 ± 0.14	0.54 ± 0.17
F		8.405	
P		0.001	

*Abbreviations*: *CI (IGTVc, IGTVs)* the degree of conformity index between IGTVc and IGTVs, *CI (IGTVc, IGTVc + s)* the degree of conformity index between IGTVc and IGTVc + s, *CI (IGTVs, IGTVc + s)* the degree of conformity index between IGTVs and IGTVc + s.

## Discussion

A majority of studies have reported that the seroma and surgical clips were used in GTV delineation and displacement measurement for EB-PBI [6-8,16]. Previous studies have reported that the target volume, seroma volume and SCS were decreasing as a function of the time from lumpectomy to the end of the radiotherapy (18–20). Kader et al. [19] reported that during postoperative Weeks 3-8 the mean seroma volume decreased from 47 to 30 cm^3^, the mean seroma clarity score was 3.4 at Weeks 3–8, 2.5 at Weeks 9–14, and 1.6 after 14 weeks. Yang et al. [20], who obtained 3 CTs (CT1, CT2, CT3) during the 6 weeks of radiotherapy for 30 patients (6 patients without CT3) underwent WBI, and found the mean TB volumes for CT1, CT2, CT3 were 42.1 cm^3^, 20.1 cm^3^, 17.0 cm^3^, respectively. How to outline the GTV based on the scattered clips in the lumpectomy cavity have not been clearly established and the volumetric nature of the cavity may not be accurately reflected. The delineation accuracy was significantly influenced by the number of clips, the delineation experience and contouring guidelines [21-24]. Kirby et al. [22] reported that the number of implanted markers would influence the accuracy in target delineation and six implanted markers are preferable in tumor bed delineation for PBI or breast boost radiotherapy. These information indicated that the optimal target volume was defined using the surgical clips, the seroma and any postoperative tissue changes.

Our ideal destination is achieving the accurate radiotherapy. The 4DCT dataset contains the spatial information for the target volume during the entire respiration cycle. The IGTV included the volume and displacement information during the whole respiration cycle, which was created by merging all the volume derived from each of the 10 respiratory phases of the 4DCT dataset [10-12]. In order to explore the advantage and disadvantage of the IGTVc + s compared with IGTVc and IGTVs for EB-PBI, we compared the variations in target size, position, DI and CI between the IGTVs defined using the seroma and/or surgical clips in current study.

Previous studies reported that the surgical clips were not always consistent with the edge of seroma and the boundary of the lumpectomy cavity [13,25]. Yang et al. [13] measured the distance between surgical clips and edge of CT-defined seroma in a coronal plane in women who have undergone wide local excision of breast cancer, and found the mean seroma edge extended beyond the clips by 0.3–0.5 cm. Goldberg et al. [25] compare the location and extent of the tumor bed as defined by surgical clips and computed tomography (CT) scans, after lumpectomy, and found the CT bed extended beyond the clips by 0–7 mm medially. The current study reported that the volume of the IGTVc + s was significantly larger than that of the IGTVc and the IGTVs. These information indicated 1) based on the size, position and displacement difference among the GTVc, GTVs and GTVc + s there were volume difference among the IGTVc, IGTVs and IGTVc + s; 2) when carrying out EB-PBI based on the IGTVc and IGTVs could increase the target missing irradiation compared with based on the IGTVc + s.

As mentioned above, the inconformity between the surgical clips and the edge of seroma could induce target volume difference between the internal gross target volumes. Meanwhile, the intrafraction GTV motion caused by respiration could influence the IGTV size. The IGTV was produced by combining these GTVs on the 10 respiratory phases of the 4DCT dataset. In this study, we measured and compared the three dimensional displacements of the GTVc, the GTVs and the GTVc + s. In the LR, AP and SI directions, the displacements were 0.9 mm, 1.05 mm and 1.20 mm for GTVc; 0.80 mm, 1.05 mm and 0.80 mm for GTVs; 0.90 mm, 1.20 mm and 1.40 mm for GTVc + s. The three dimensional displacements of the GTVc + s were greater than the GTVc and GTVs (16). This information indicated that the internal gross target volume included the information about the GTV size and the displacement, and the displacements have contribution to the volume difference among the internal gross target volumes.

In order to estimate the volume of missing irradiation in the event of performing treatment planning based on IGTVc + s, we introduced the degree of inclusion of IGTVc + s in IGTVx [DI (IGTVc + s in IGTVx)], 1-DI (IGTVc + s in IGTVx) then represented the proportion that missing irradiation accounts for IGTVx (IGTVc and IGTVs). Table 3 present a information that assuming the IGTVc + s as the ideal target, there would be 29% of (mean) missing irradiation for IGTVc and 43% (mean) for IGTVs. In addition, our study reported a mean of CI between IGTVc and IGTVs, IGTVc and IGTVc + s, IGTVs and IGTVc + s were range from 0.4-0.63. These informations indicated that there were obvious spatial mismatch between the internal gross target volumes defined using the seroma and surgical clips.

Park et al. [26] compared the relative position of the center of mass (COM) of the fiducials with the geometric center of the seroma, and found the average position of the geometric seroma relative to the fiducial COM pretreatment compared with posttreatment was 1 mm ± 1 mm. Similar results were acquired in the current study, the displacements of the COM between IGTVc and IGTVs, IGTVc and IGTVc + s, IGTVs and IGTVc + s were (0.66 ± 0.31), (0.42 ± 0.36), (0.32 ± 0.17), respectively. And the distances of the COM were negatively correlated with the DI and CI between the IGTVs. Landis et al. [27] examined the interobserver variability in TB delineation for PBI among radiation oncologists, and found a mean overlap of 57%, 68% for the PTV, and the center of mass of the volume was displaced by a median of 6.9 mm and 3.9 mm, respectively, for cavities with a cavity visualization score of 2 or 3. These information indicated that the overlapping relation between two internal gross target volumes became poor as the distance of the COM between two internal gross target volumes increased.

It should be noted that we contouring the GTV on each of the 10 respiratory phases of the 4DCT dataset and combining these GTVs to produce IGTV in this study. However, the delineation variation and artifacts during the 10 CT could influence the accuracy of the IGTV. We compared the coefficient of variation (CV) induced by the delineation and the respiration, and found the CV induced by deformation and displacement of the lumpectomy cavity during the respiration cycle was larger than that induced by delineation variation (0.08 vs 0.04, *p* = 0.002).

## Conclusion

We have shown the internal gross target volume could ensure adequately coverage of the moving target within the radiation field without missing irradiation. There was spatial mismatch among the internal gross target volumes delineated based on the surgical clips and/or the seroma, and the overlapping relation between two internal gross target volumes became poor as the distance of the COM between two internal gross target volumes increased. IGTVc + s provided the best overall representation of the ‘true’ moving GTV, though it is defined using the seroma and surgical clips.

## Abbreviations

BCS: Breast-conserving surgery; EB-PBI: External-beam partial breast irradiation; PBI: Partial breast irradiation; IGTV: Internal gross tumor volume; ITV: Internal target volume; 4DCT: Four-dimensional computed tomography; COM: Center of mass; LR: Left-right; AP: Anterior-posterior; SI: Senior-inferior; DI: Degree of inclusion; CI: Conformity index; TPS: Treatment planning system; 3DCRT: Three-dimensional conformal radiotherapy; IMRT: Intensity-modulated radiotherapy.

## Competing interests

The authors declare that they have no competing interests.

## Authors’ contributions

YD, JBL participated in the study design, contributed to the data collection, and draft the manuscript. WW, SZW, JZW and ZFM, made important contributions in the design of the study and in revising the content. MX and QS contributed in collecting and analyzing data. All authors read and approved the final manuscript.
